# Tautomerism of Guanine Analogues

**DOI:** 10.3390/biom10020170

**Published:** 2020-01-22

**Authors:** Jakub Radek Štoček, Martin Dračínský

**Affiliations:** Institute of Organic Chemistry and Biochemistry, Czech Academy of Sciences, Flemingovo nám. 2, 166 10 Prague, Czech Republic

**Keywords:** nucleic acids, tautomerism, NMR spectroscopy, DFT calculations

## Abstract

Tautomerism of nucleic acid (NA) bases is a crucial factor for the maintenance and translation of genetic information in organisms. Only canonical tautomers of NA bases can form hydrogen-bonded complexes with their natural counterparts. On the other hand, rare tautomers of nucleobases have been proposed to be involved in processes catalysed by NA enzymes. Isocytosine, which can be considered as a structural fragment of guanine, is known to have two stable tautomers both in solution and solid states. The tautomer equilibrium of isocytosine contrasts with the remarkable stability of the canonical tautomer of guanine. This paper investigates the factors contributing to the stability of the canonical tautomer of guanine by a combination of NMR experiments and theoretical calculations. The electronic effects of substituents on the stability of the rare tautomers of isocytosine and guanine derivatives are studied by density functional theory (DFT) calculations. Selected derivatives are studied by variable-temperature NMR spectroscopy. Rare tautomers can be stabilised in solution by intermolecular hydrogen-bonding interactions with suitable partners. These intermolecular interactions give rise to characteristic signals in proton NMR spectra, which make it possible to undoubtedly confirm the presence of a rare tautomer.

## 1. Introduction

Nucleic acid bases are responsible for maintaining and translating genetic information in organisms. Hydrogen bonding is the key interaction for the recognition of nucleobases during the replication and translation processes.

In their seminal work, Watson and Crick emphasised the importance of the tautomerism of nucleic acid (NA) bases for DNA structures; only canonical tautomers of NA bases can form hydrogen-bonded complexes with their natural counterparts [1]. A tautomeric change modifies the hydrogen-bonding pattern of the nucleobase (a hydrogen-bond acceptor to a hydrogen-bond donor, and vice versa). Tautomeric modifications of DNA bases are, therefore, undesirable, as they can lead to errors in replication and transcription [2,3,4,5]. On the other hand, modifications of tautomeric forms have been speculated to play an important role in the catalysis and binding by RNA enzymes and aptamers [6]. For example, minor tautomeric and/or ionised forms of guanine have been proposed in the catalytic site of self-cleaving ribozymes [7].

**Figure 1:**
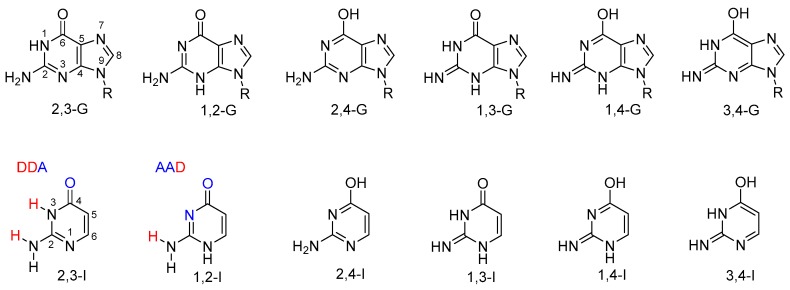
Possible tautomers of 9-substituted guanine (**top**) and analogous tautomers of isocytosine (**bottom**). Hydrogen-bonding donors (D) and acceptors (A) are highlighted in the structures of the two major isocytosine tautomers.

Guanine is one of the nucleobases. Possible tautomeric forms of 9-substituted guanine are shown in Figure 1. Tautomerism of guanine has been investigated theoretically in many studies; see, for example, References [8,9,10,11,12,13] and the references therein. Four guanine tautomers have been detected in the gas phase [14,15]. Note, however, that these tautomers include two 7*H* forms, which are not possible for the 9-substituted guanine found in nucleic acids. Despite numerous experimental studies, there is no clear evidence of the presence of a rare tautomer of the 9-substituted or 9*H* guanine base in solution; see, e.g., References [16,17,18,19,20].

In contrast to guanine, its close analogue isocytosine (a structural fragment of guanine) is known to have two stable tautomers (2,3-I and 1,2-I; Figure 1). The following notation of isocytosine tautomers has previously been used and is employed throughout this work; it indicates the atoms to which the exchangeable hydrogen atoms are attached. For consistency, the guanine tautomers are named analogously. Thus, the canonical tautomer of guanine is referred to as 2,3-G in this work. The two isocytosine tautomers offer different bonding patterns for potential intermolecular hydrogen bonding: 1,2-I has an acceptor–acceptor–donor pattern (AAD; Figure 1), whereas 2,3-I has a donor–donor–acceptor pattern (DDA). These two tautomers are, therefore, complementary and can form a dimer interconnected by three hydrogen bonds analogous to those found in the guanine–cytosine base pair. Both tautomers are found in a 1:1 ratio in solid isocytosine, where the basic structural motif contains the hydrogen-bonded dimer of the two isocytosine tautomers [21,22,23]. This isocytosine dimer has also been observed in solution [22,24,25].

The tautomerism of isocytosine has also been studied by density functional theory (DFT) computations, which have confirmed that 1,2-I and 2,3-I have the lowest energy (the 2,3-I tautomer is the most stable) [26]. Two other tautomers (2,4-I and 1,3-I) also had relatively low energy (less than 28 kJ/mol above the most stable tautomer). On the other hand, 1,4-I and 3,4-I tautomers had energy at least 60 kJ/mol higher than the most stable tautomer.

We have recently demonstrated that the formation of an intermolecular complex with a suitable hydrogen-bonding partner can stabilise a selected isocytosine tautomer [24]. A compound with an AAD hydrogen-bonding pattern can form a stable intermolecular complex with the 2,3-I tautomer, whereas a compound with a DDA pattern can provide complexes with the 1,2-I tautomer. Therefore, the additions of these compounds can change the relative ratio of 1,2-I and 2,3-I tautomers in solution.

Here, we investigate the factors contributing to the stability of the canonical guanine tautomer in contrast with the tautomeric equilibrium of isocytosine by a combination of NMR experiments and theoretical calculations. First, we screened the effects of substituents on the relative energy of isocytosine and guanine tautomers. Next, we performed NMR experiments, which confirmed the remarkable stability of the canonical guanine tautomer and demonstrated the stabilisation of rare tautomers of some isocytosine derivatives by intermolecular interactions.

## 2. Materials and Methods

The preparations of compounds **18** and **T** were described in References [24] and [27], respectively. Compounds **1**, **7**, **10**, **16** and diaminopyridine (**DAP**) were purchased from Sigma Aldrich and used without purification. The concentration of all samples for NMR experiments was 10 mM.

Variable-temperature ^1^H-NMR spectra were recorded on a Bruker 500 MHz NMR spectrometer (^1^H at 500.0 MHz) in a 3:1 mixture of dimethylformamide (DMF)-*d*_7_ and CD_2_Cl_2_ (referenced to the solvent signal δ = 2.75). The assignment of the signals was based on analogy with previously published data [25].

The studied structures were subjected to geometry optimisation at the DFT level using the B3LYP functional [28,29] with the standard 6-311++G(2df,2pd) basis set and the polarisable continuum model used for implicit DMF solvation [30,31]. Empirical dispersion correction GD3 was used in all calculations [32]. An initial screening of relative tautomer energies was performed with a smaller basis set, 6-31g(d,p); these energies are reported in the SI. To simplify the calculations, the alkyl chain of compound **T** was substituted by a methyl group. The vibrational frequencies and free energies were calculated for all of the optimised structures, and the stationary–point (minimum) character was thus confirmed. The Gaussian16 program package was used throughout this study [33]. No corrections of basis set superposition error (BSSE) were included in the computations of intermolecular complexes, because the large basis set used in the calculations should minimise this error. A counterpoise correction method applied for the isocytosine dimer provided an estimation of the BSSE of 2.7 kJ/mol.

## 3. Results and Discussion

### 3.1. Computation—Monomers

**Figure 2:**
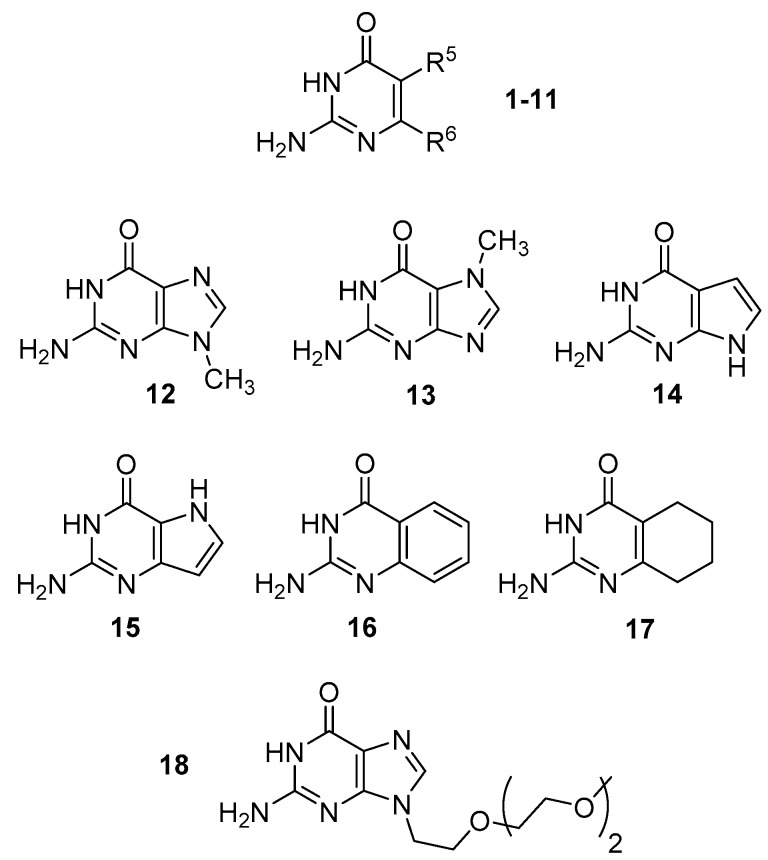
The structure of compounds **1**–**18**.

We have calculated the relative energies of the four low-energy tautomers (2,3-I; 1,2-I; 2,4-I and 1,3-I) of a series of isocytosine derivatives with various substituents in positions 5 and 6 (Table 1 and Figure 2). Electron-accepting NO_2_ and CF_3_ groups, electron-donating NH_2_ and CH_3_ groups and the sterically demanding *tert*-butyl group were used in the series. The 2,3-I tautomer has the lowest energy in all cases. However, the relative energy and relative order of the remaining tautomers are strongly substituent-dependent.

The second-most stable tautomer is always either 1,2-I or 2,4-I (enol). The relative energies of the 1,2-I tautomer substituted in position 5 strongly depend on the electronic nature of the substituent; the electron-donating amino group (compound **4**) stabilises the 1,2-I tautomer (with the relative energy 7.4 kJ/mol lower than for isocytosine **1**), while the electron-accepting nitro group destabilises this tautomer (with the relative energy 11.0 kJ/mol higher than for isocytosine **1**). Interestingly enough, the 1,2-I tautomer of the derivatives substituted in position 6 is destabilised by both electron-accepting and electron-donating groups, except alkyl groups. These calculations agree with the previous observation that the 2,3-I tautomer of compound **9** is strongly preferred in cocrystallisation with compounds containing complementary functional groups, whereas both 1,2-I and 2,3-I tautomers of compound **7** are formed in cocrystals with suitable partners [34].

The enol tautomer (2,4-I) is relatively stable in the case of isocytosine **1** and 6-nitroisocytosine **11**, where it is only 20 kJ/mol less stable than the lowest-energy 2,3-I tautomer. In most cases, the imino tautomer 1,3-I is the least stable of the investigated tautomers (with the energy at least 21 kJ/mol higher than that of the 2,3-I tautomer). The imino tautomer is particularly unstable for derivatives substituted with electron-accepting or strongly electron-donating groups in position 6.

We have also computationally investigated a series of bicyclic compounds (**12**–**17**) that includes guanine methylated in position 7 or 9, its 7-deaza and 9-deaza derivatives and benzo and tetrahydrobenzo derivatives (Table 2). Like in the case of the isocytosine derivatives discussed above, the 2,3-tautomer is always the most stable. The 1,2-tautomer is relatively stable for 9-deaza guanine **15** and for benzo and tetrahydrobenzo derivatives **16** and **17**. The enol and imino tautomers of these bicyclic compounds always have relatively high energy.

The instability of rare tautomers of 9-methylguanine and 7-deazaguanine are particularly interesting. These two derivatives are the only ones in the investigated series where all rare tautomers have energy at least 33 kJ/mol higher than the canonical 2,3-tautomer.

### 3.2. Experiments

Isocytosine in solution forms a dimer of hydrogen-bonded 1,2-I and 2,3-I tautomers upon cooling [24,25]. This means that the free-energy gain connected with the dimer formation is larger than the free-energy penalty needed for the formation of the less stable 1,2-I tautomer. It is reasonable to expect that the free-energy change upon the formation of a 1,2-I–2,3-I dimer will also be similar for substituted isocytosine, because the number and character of the hydrogen bonds will be the same. Therefore, whether a dimer is formed is mostly controlled by the relative energy of the minor 1,2-I tautomer.

We have selected a series of isocytosine and guanine derivatives differing in the energy of the 1,2-I tautomer and performed NMR experiments at temperatures ranging from 295 to 175 K. The series includes isocytosine **1**, 6-methylisocytosine **7**, 6-trifluoromethylisocytosine **10**, benzoderivative **16** and alkylated guanine **18** (Figure 2, the oligoethoxy chain has been used to improve the solubility).

**Figure 3:**
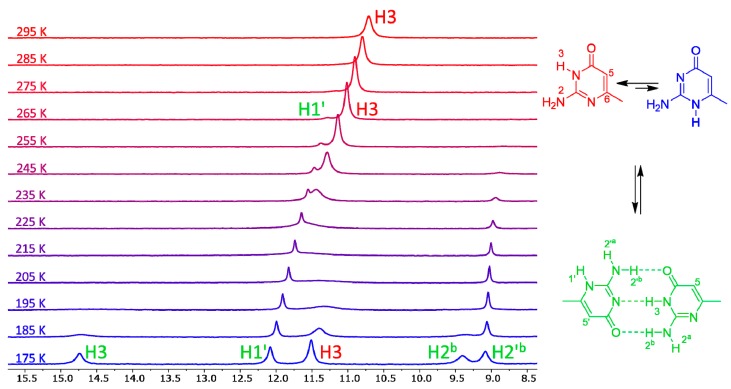
The low-field regions of variable-temperature ^1^H-NMR spectra of compound **7** in a 3:1 mixture of dimethylformamide (DMF)-*d*_7_ and CD_2_Cl_2_. Signals of imino hydrogens H1′ and H3, together with signals of amino hydrogens involved in intermolecular hydrogen bonds, appear in this spectral region. Another spectral region is shown in the SI (Figure S1).

The variable-temperature NMR spectra of compound **7** are shown in Figure 3. This compound has one of the lowest calculated energy differences among the monocyclic isocytosine derivatives between the major 2,3-I and minor 1,2-I tautomers. At room temperature, it is possible to observe one set of signals corresponding to the 2,3-I tautomer (or to a fast-exchanging mixture of both tautomers with high excess of the 2,3-I tautomer). However, new signals appear in the spectra at 275 K and below. These new signals correspond to the dimer of the two tautomers. This dimer formation is observed, although the solvent mixture used for the experiments favours the monomers; dimethylformamide is a good hydrogen-bond acceptor and can solvate the amino and imino hydrogens of the monomers, which are not accessible by the solvent when the dimer is formed.

The signals corresponding to the dimer of compound **1** appeared at a slightly lower temperature of 255 K (Figure S2), which is in agreement with the fact that the calculated energy difference between the two tautomers is slightly higher for this compound. The minor tautomer of compound **10** is significantly less stable than that of compounds **1** and **7**, and we have not observed any signals of the dimer of this compound even at the lowest temperature of 175 K (Figure S3).

Similarly, we have not observed any formation of the 1,2-G–2,3-G dimer of guanine derivative **18** (Figure S4), which was expected, because the calculated relative energy of the 1,2-G tautomer was the highest of all the investigated compounds.

The calculated relative energy of the 1,2-I tautomer of benzo derivative **16** was even lower than that of compound **7**, and we have observed signals corresponding to the dimer already at 285 K (Figure S5). However, the signals are broad at this temperature and not well-separated from the signals of the 2,3-I monomer (Figure S5).

We have previously shown that intermolecular interactions with suitable hydrogen-bonding partners can stabilise the minor tautomer (1,2-I) of isocytosine [24]. Here, we have performed variable-temperature experiments with mixtures of compounds containing an isocytosine derivative that might form a rare tautomer with a compound that can form three hydrogen bonds with this tautomer.

**Figure 4:**
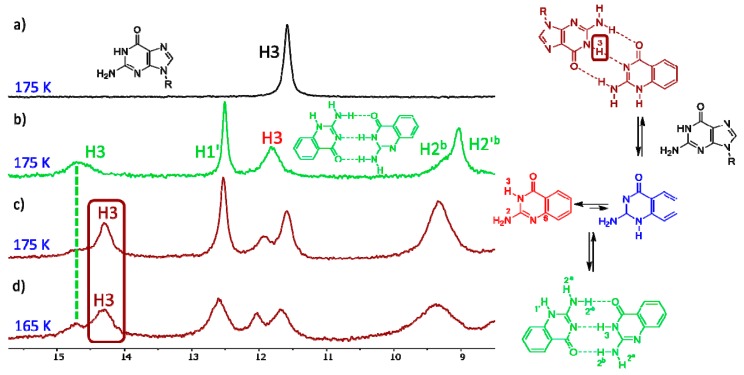
The low-field region of the ^1^H-NMR spectra of compound **18** (**a**), compound **16** (**b**) and their 1:1 mixture (**c**,**d**).

When guanine derivative **18** is added to the solution of isocytosine derivative **16**, a new signal in the high chemical shift range appears. This clearly indicates the formation of a hydrogen-bonding complex between these two molecules (Figure 4) and confirms the stabilisation of the 1,2-I tautomer of compound **16** by intermolecular interactions. Similarly, the formation of an intermolecular complex between the 1,2-I tautomer of 6-methylisocytosine **7** and guanine derivative **18** has been observed (Figure S6). On the other hand, no complex formation has been observed in the mixture of trifluoromethyl derivative **10** and guanine **18** (Figure S7). Clearly, the stabilisation of the intermolecular complex cannot overcome the large free-energy penalty needed for the formation of the 1,2-I tautomer of compound **10**.

**Figure 5:**
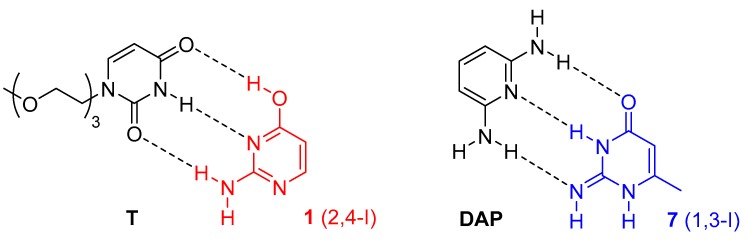
The expected structure of the enol tautomer 2,4-I of isocytosine **1** in a complex with thymine derivative **T** (**left**), and the structure of the imino tautomer 1,3-I of compound **7** in a complex with diaminopyridine (**DAP**, **right**). These complexes were not observed.

We also speculated that a thymine derivative might stabilise the enol (2,4-I) tautomer of isocytosine (Figure 5), because this isocytosine tautomer has relatively low energy (Table 1). Similarly, diaminopyrimidine (**DAP**) might stabilise the imino tautomer (1,3-I) of compound **7** by the formation of an intermolecular complex with three hydrogen bonds. However, we have only observed the formation of isocytosine dimers in the mixture of compound **1** with **T**, which means that the energy gain produced by the complex formation of the enol tautomer of isocytosine with thymine could not overcome the energy barrier of the enol 2,4-I tautomer (Figure S8). Similarly, only dimers of compound **7** have been observed in the mixture of compound **7** with **DAP** (Figure S9).

### 3.3. Computations—Complexes

To rationalise the observations of intermolecular complexes further, we have performed geometry optimisation of selected dimers of isocytosine and guanine derivatives (1,2-I–2,3-I) and of complexes of selected minor tautomers with the hydrogen-bonding partners used in the NMR experiments. Table 3 summarises the calculated stabilisation energies (*E*_stabil_) of these dimers and complexes. These energies were calculated as the difference between the energy of the dimer/complex and the energies of the most stable tautomer of its components. For comparison, the table also includes complexation energies (*E*_complex_), which were calculated as the difference between the energy of the dimer/complex and the energies of its components in the same tautomeric form as in the dimer/complex. The complexation energies thus reflect the strength of intermolecular hydrogen-bonding interactions, whereas the stabilisation energies include the energy penalty needed for the formation of a less stable tautomer. Additionally, note that solvent effects are only implicitly involved in the calculations using a polarisable continuum model. The real solvation of the molecules in solution will disfavour the dimer/complex formation, because dimethylformamide solvates the monomers well.

The computations of dimer stabilisation energies nicely reflect the experimental findings described above. The greatest stabilisation was calculated for the dimer of compound **16**, followed by the dimers of compounds **7** and **1**, which means that the stabilisation energies have the same order as the appearance of the dimer signals in the spectra. The dimer of compound **10** is 20 kJ/mol less stable than the dimers of compounds **1**, **7** and **16**, which is in agreement with the fact that we did not observe the formation of this dimer at all. The lowest magnitude of stabilisation energy was found for the dimer of guanine derivative **12** (reflecting the highest relative energy of the 1,2-G tautomer).

The computations have also confirmed that guanine in its canonical tautomer can stabilise 1,2-I tautomers of compound **7** or **16**. On the other hand, the stabilisation of the complex of the isocytosine enol tautomer (2,4-I) with thymine is almost 20 kJ/mol lower than the stabilisation of the isocytosine dimer, which explains why only the dimer has been observed experimentally and no traces of the 2,4-I tautomer have been found. Similarly, the stabilisation of the complex of the isocytosine imino tautomer 1,3-I with diaminopyridine (**DAP**) is too low to enable the observation of this complex.

## 4. Conclusions

We have studied the tautomeric equilibria of isocytosine and guanine derivatives. Guanine has a strong preference for its canonical tautomer that is found in DNA. Isocytosine is a structural fragment of guanine, but it has significantly lower relative energy of its minor tautomeric forms, particularly the 1,2-I tautomer. We have investigated the factors contributing to the remarkable differences between the stability of minor tautomers of guanine and isocytosine.

The DFT calculations have revealed that the 1,2-I tautomer is stabilised by electron-donating groups in position 5 of isocytosine and destabilised by electron-accepting groups in this position. The same tautomer is destabilised by both electron-accepting and strongly electron-donating groups in position 6. All minor tautomers of 9-methylguanine (**12**) have very high-calculated relative energies. Interestingly, 7-methylguanine (**13**) has significantly lower relative energy of the 1,2-tautomer than 9-methylguanine.

The variable-temperature NMR experiments have demonstrated that isocytosine and its derivatives with low relative energy of the 1,2-I tautomer form a dimer in solution, where the 2,3-I form is bound to the 1,2-I form by three intermolecular hydrogen bonds. The DFT calculations of the relative energy of the 1,2-I tautomer correlate very well with the observed stability of the dimers. The addition of guanine derivative **18** to the solution of a compound with a low-energy 1,2-I tautomer leads to the formation of an intermolecular complex consisting of the 2,3-G tautomer of guanine and the 1,2-I tautomer of the isocytosine derivative.

We also speculated that other minor tautomers of isocytosine might be stabilised by intermolecular interactions with suitable hydrogen-bonding partners. However, we have not observed any intermolecular complexes between thymine derivative **T** and isocytosine, or between diaminopyridine and 6-methylisocytosine (**7**). The DFT calculations have revealed that although the suggested intermolecular complexes also have three intermolecular hydrogen bonds, the magnitude of the complexation energy is significantly lower than in the complexes containing the 1,2-I tautomer.

Importantly, we have not observed any noncanonical tautomer of guanine derivative **18** in any of the experiments. Furthermore, the relative energies of all minor tautomers of guanine are very high (at least 33 kJ/mol above the canonical tautomer); they are, in most cases, the highest among all the studied compounds. The instability of rare tautomers may have played a role in the natural selection of nucleobases suitable for the reliable storage of genetic information. The extraordinary stability of the canonical guanine tautomer is a key factor in high-fidelity replication, transcription and translation of the genetic code. Furthermore, in view of these results, the involvement of rare guanine tautomers in processes catalysed by nucleic acids seems to be improbable.

## Figures and Tables

**Table 1:** The relative energies (kJ/mol) of four tautomers of compounds **1**–**11** (Figure 2) calculated at the B3LYP/6-311++G(2df,2pd) level.

	R^5^	R^6^	2,3-I (keto)	1,2-I (keto)	2,4-I (enol)	1,3-I (imino) ^1^
**1**	H	H	0.0	13.9	20.7	23.3
**2**	CH_3_	H	0.0	12.0	30.5	23.8
**3**	*t*-butyl	H	0.0	12.8	22.0	22.3
**4**	NH_2_	H	0.0	6.5	25.9	29.6
**5**	CF_3_	H	0.0	16.3	24.3	28.7
**6**	NO_2_	H	0.0	24.9	24.6	34.8
**7**	H	CH_3_	0.0	12.6	28.2	21.3
**8**	H	*t*-butyl	0.0	12.8	27.8	22.3
**9**	H	NH_2_	0.0	34.8	28.0	36.7
**10**	H	CF_3_	0.0	32.3	29.0	45.3
**11**	H	NO_2_	0.0	32.5	20.0	49.1

^1^ The energies of both rotamers of the imino group have been calculated, the lower of which is presented here. The energies of both rotamers are given in the SI.

**Table 2:** The relative energies (kJ/mol) of four tautomers of bicyclic compounds **12**–**17** (Figure 2) calculated at the B3LYP/6-311++G(2df,2pd) level.

	2,3-I (keto)	1,2-I (keto)	2,4-I (enol)	1,3-I (imino) ^1^
**12**	0.0	41.0	33.8	49.0
**13**	0.0	17.2	34.3	32.6
**14**	0.0	38.5	38.3	45.1
**15**	0.0	11.5	48.7	27.3
**16**	0.0	8.5	46.8	20.1
**17**	0.0	10.8	30.6	21.0

^1^ The energies of both rotamers of the imino group have been calculated, the lower of which is presented here. The energies of both rotamers are given in the SI.

**Table 3:** The calculated complexation and stabilisation energies (kJ/mol) of isocytosine and guanine derivatives.

Dimer/Complex Structure	*E*_complex_	*E*_stabil_
**1**(2,3-I) + **1**(1,2-I)	−79.9	−66.0
**7**(2,3-I) + **7**(1,2-I)	−80.1	−67.5
**16**(2,3-I) + **16**(1,2-I)	−77.3	−68.8
**10**(2,3-I) + **10**(1,2-I)	−79.5	−47.2
**12**(2,3-G) + **12**(1,2-G)	−78.1	−37.2
**7**(1,2-I) + **12**(2,3-G)	−79.2	−66.6
**16**(1,2-I) + **12**(2,3-G)	−78.1	−69.7
**1**(2,4-I) + **T**	−68.7	−47.9
**1**(1,3-I) + **DAP**	−55.0	−32.6

## Supplementary Materials

The following are available online at https://www.mdpi.com/2218-273X/10/2/170/s1: Table S1. Relative energies (kJ/mol) of four tautomers of compounds **1**–**11** calculated at the B3LYP/6-311++G(2df,2pd) level. Table S2. Relative energies (kJ/mol) of four tautomers of bicyclic compounds **12**–**17** calculated at the B3LYP/6-311++G(2df,2pd) level. Table S3. Relative energies (kJ/mol) of four tautomers of compounds **1**–**11** calculated at the B3LYP/6-31+G(d,p) level. Table S4. Relative energies (kJ/mol) of four tautomers of bicyclic compounds **12**–**17** calculated at the B3LYP/6-31+G(d,p) level. Figure S1. H5 and NH_2_ regions of variable-temperature ^1^H-NMR spectra of compound **7** in a 3:1 mixture of DMF-*d*_7_ and CD_2_Cl_2_. Figure S2. Low-field region of variable-temperature ^1^H-NMR spectra of compound **1** in a 3:1 mixture of DMF-*d*_7_ and CD_2_Cl_2_. Figure S3. Low-field region of variable-temperature ^1^H-NMR spectra of compound **10** in a 3:1 mixture of DMF-*d*_7_ and CD_2_Cl_2_. Figure S4. Low-field region of variable-temperature ^1^H-NMR spectra of compound **18** in a 3:1 mixture of DMF-*d*_7_ and CD_2_Cl_2_. Figure S5. Low-field region of variable-temperature ^1^H-NMR spectra of compound **16** in a 3:1 mixture of DMF-*d*_7_ and CD_2_Cl_2_. Figure S6. Low-field region of ^1^H-NMR spectra of compound **18** (a), compound **7** (b) and their 1:1 mixture (c). Figure S7. Low-field region of ^1^H-NMR spectra of compound **18** (a), compound **10** (b) and their 1:1 mixture (c). Figure S8. Low-field region of ^1^H-NMR spectra of compound **1** (a), compound **T** (b) and their 1:1 mixture (c). Figure S9. Low-field region of ^1^H-NMR spectra of compound **7** (a), compound **DAP** (b) and their 1:1 mixture (c).
