# Supplementary material for: Tautomerism of Guanine Analogues
Source: Biomolecules. 2020 Jan 22;10(2):170. doi: 10.3390/biom10020170 (PMC7072560; doi:10.3390/biom10020170)
Supplement: Supplementary file 1 [file biomolecules-10-00170-s001.pdf]

Tautomerism of guanosine analogues

Jakub Radek Štoček[1] and Martin Dračínský[1,*]

[1] Institute of Organic Chemistry and Biochemistry, Czech Academy of Sciences, Flemingovo nám. 2, 166 10, Prague, Czech Republic.

* Correspondence: dracinsky@uochb.cas.cz

Supporting Information

Table S1. Relative energies (kJ/mol) of four tautomers of compounds **1–11** calculated at B3LYP/6-311++G(2df,2pd) level.

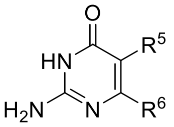

	R^5	R^6	2,3-I (keto)	1,2-I (keto)	2,4-I (enol)	1,3-I (imino)[a]	1,3-I (imino)[b]
**1**	H	H	0.0	13.9	20.7	23.3	24.4
**2**	CH_3	H	0.0	12.0	30.5	23.8	24.5
**3**	*t*-butyl	H	0.0	12.8	22.0	22.3	23.1
**4**	NH_2	H	0.0	6.5	25.9	29.8	29.6
**5**	CF_3	H	0.0	16.3	24.3	28.7	30.3
**6**	NO_2	H	0.0	24.9	24.6	34.8	37.5
**7**	H	CH_3	0.0	12.6	28.2	21.3	22.4
**8**	H	*t*-butyl	0.0	12.8	27.8	22.3	23.6
**9**	H	NH_2	0.0	34.8	28.0	36.7	38.8
**10**	H	CF_3	0.0	32.3	29.0	45.3	46.6
**11**	H	NO_2	0.0	32.5	20.0	49.1	49.8

[a]Imino hydrogen heading towards H3; [b]imino hydrogen heading towards N1

Table S2. Relative energies (kJ/mol) of four tautomers of bicyclic compounds **12–17** calculated at B3LYP/6-311++G(2df,2pd) level.

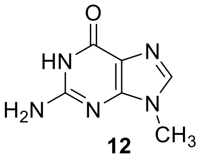
12
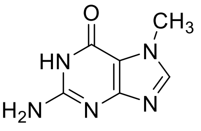
13
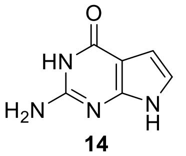
14
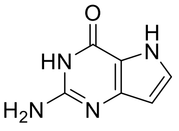
15
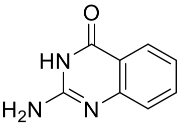
16
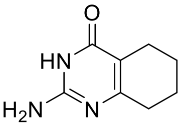
17

	2,3-I (keto)	1,2-I (keto)	2,4-I (enol)	1,3-I (imino)[a]	1,3-I (imino)[b]
**12**	0.0	41.0	33.8	49.0	50.1
**13**	0.0	17.2	34.3	32.8	32.6
**14**	0.0	38.5	38.3	45.1	45.9
**15**	0.0	11.5	48.7	27.5	27.3
**16**	0.0	8.5	46.8	20.4	20.1
**17**	0.0	10.8	30.6	21.0	21.7

[a]Imino hydrogen heading towards H3; [b]imino hydrogen heading towards N1

Table S3. Relative energies (kJ/mol) of four tautomers of compounds **1–11** calculated at B3LYP/6-31+G(d,p) level.

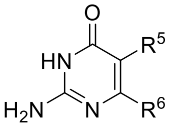

	R^5	R^6	2,3-I (keto)	1,2-I (keto)	2,4-I (enol)	1,3-I (imino)[a]	1,3-I (imino)[b]
**1**	H	H	0.00	21.5	15.5	26.2	27.8
**2**	CH_3	H	0.00	19.2	29.0	26.9	27.9
**3**	*t*-butyl	H	0.00	19.1	33.2	25.58	26.7
**4**	NH_2	H	0.00	12.9	22.1	33.1	32.8
**5**	CF_3	H	0.00	20.2	22.7	30.5	32.6
**6**	NO_2	H	0.00	32.3	21.2	37.0	40.6
**7**	H	CH_3	0.00	19.5	25.0	24.0	25.5
**8**	H	*t*-butyl	0.00	21.4	24.5	26.4	28.2
**9**	H	NH_2	0.00	41.1	23.4	39.4	42.0
**10**	H	CF_3	0.00	36.0	26.3	44.3	45.9
**11**	H	NO_2	0.00	37.6	15.4	50.0	51.0

[a]Imino hydrogen heading towards H3; [b]imino hydrogen heading towards N1

Table S4. Relative energies (kJ/mol) of four tautomers of bicyclic compounds **12–17** calculated at B3LYP/6-31+G(d,p) level.

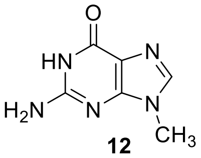

12

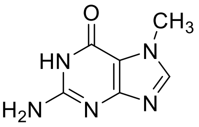

13

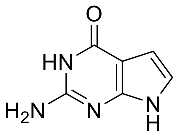

14

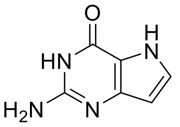

15

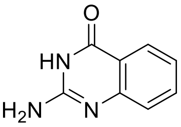

16

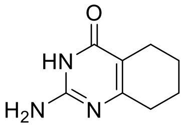

17

	2,3-I (keto)	1,2-I (keto)	2,4-I (enol)	1,3-I (imino)[a]	1,3-I (imino)[b]
**12**	0.0	49.6	30.5	53.1	54.4
**13**	0.0	21.2	31.2	34.3	34.0
**14**	0.0	46.4	36.2	48.8	49.8
**15**	0.0	17.8	47.6	30.1	29.8
**16**	0.0	14.7	48.6	23.0	22.8
**17**	0.0	17.4	29.6	24.0	25.0

[a]Imino hydrogen heading towards H3; [b]imino hydrogen heading towards N1

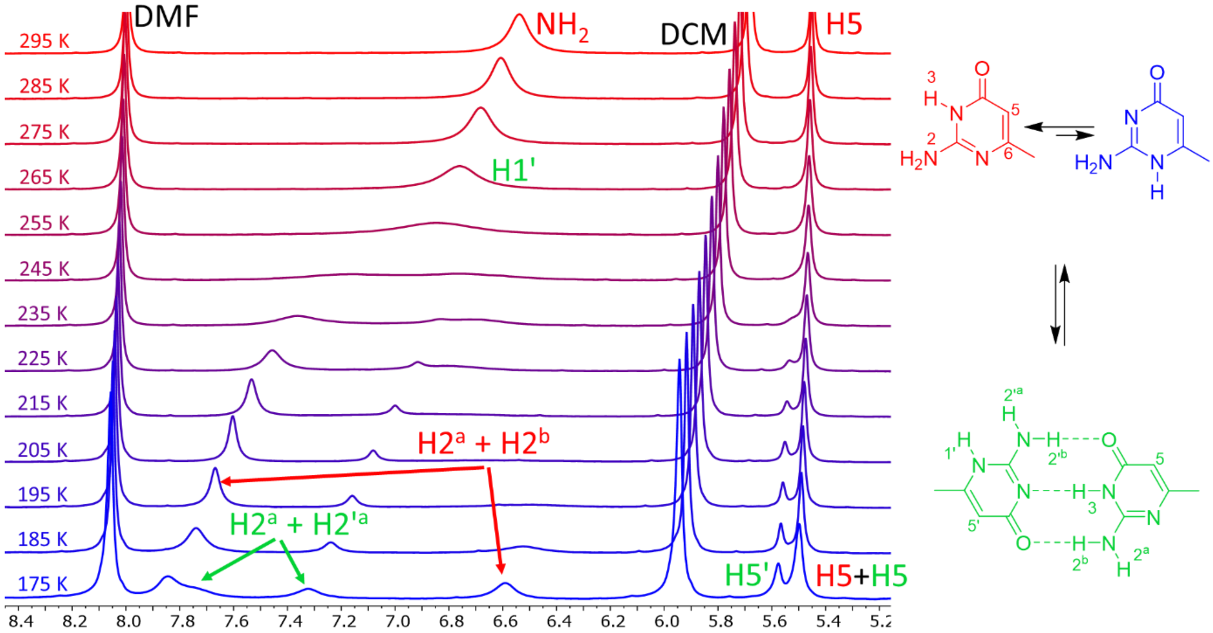

Figure S1. H5 and NH_2 region of variable-temperature ^1H NMR spectra of compound **7** in a 3:1 mixture of DMF-d_7 and CD_2Cl_2.

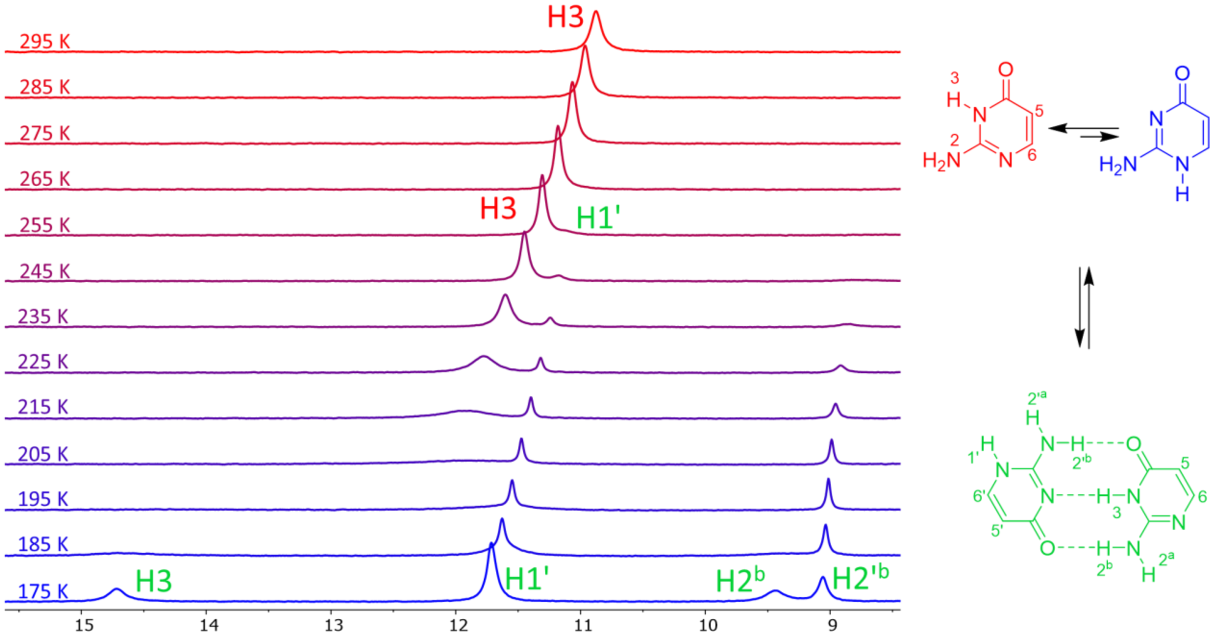

Figure S2. Low-field region of variable-temperature ^1H NMR spectra of compound **1** in a 3:1 mixture of DMF-d_7 and CD_2Cl_2.

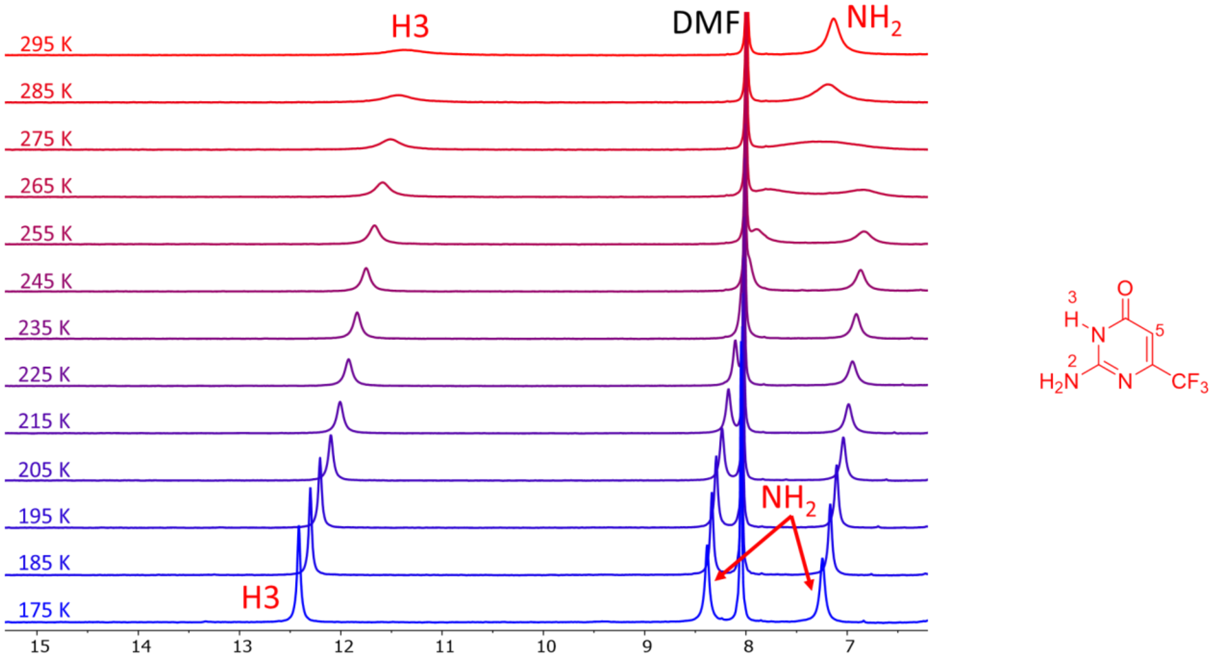

Figure S3. Low-field region of variable-temperature ^{1}H NMR spectra of compound **10** in a 3:1 mixture of DMF-*d*7 and CD_2Cl_2.

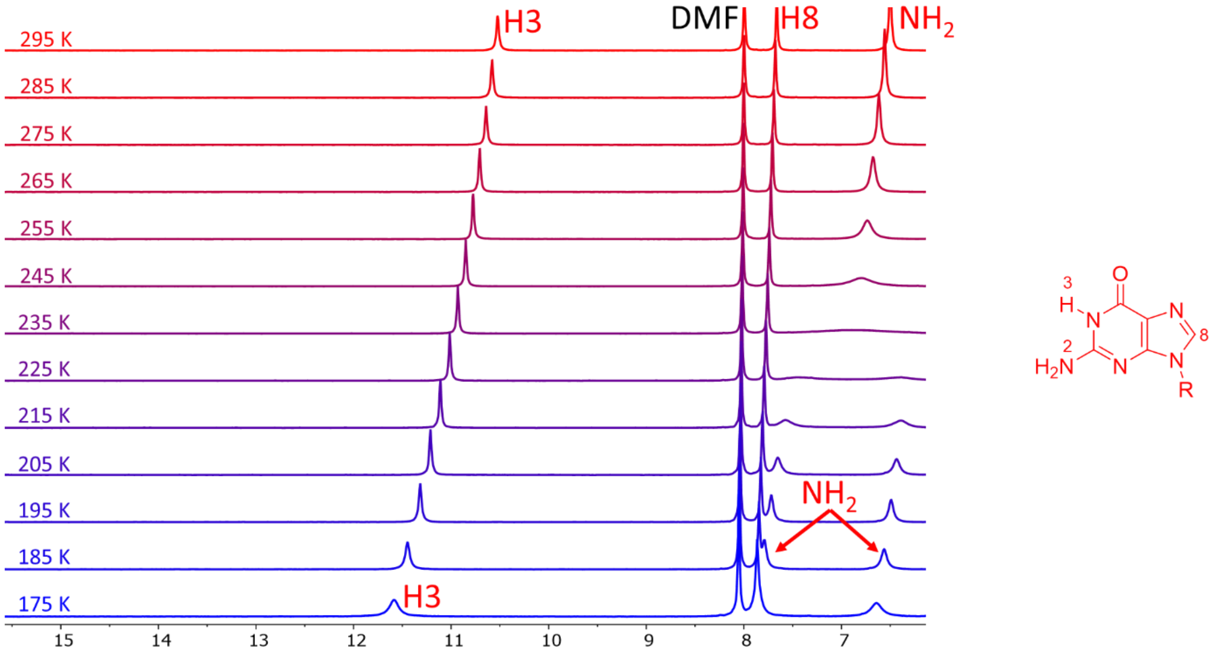

Figure S4. Low-field region of variable-temperature ^{1}H NMR spectra of compound **18** in a 3:1 mixture of DMF-*d*7 and CD_2Cl_2.

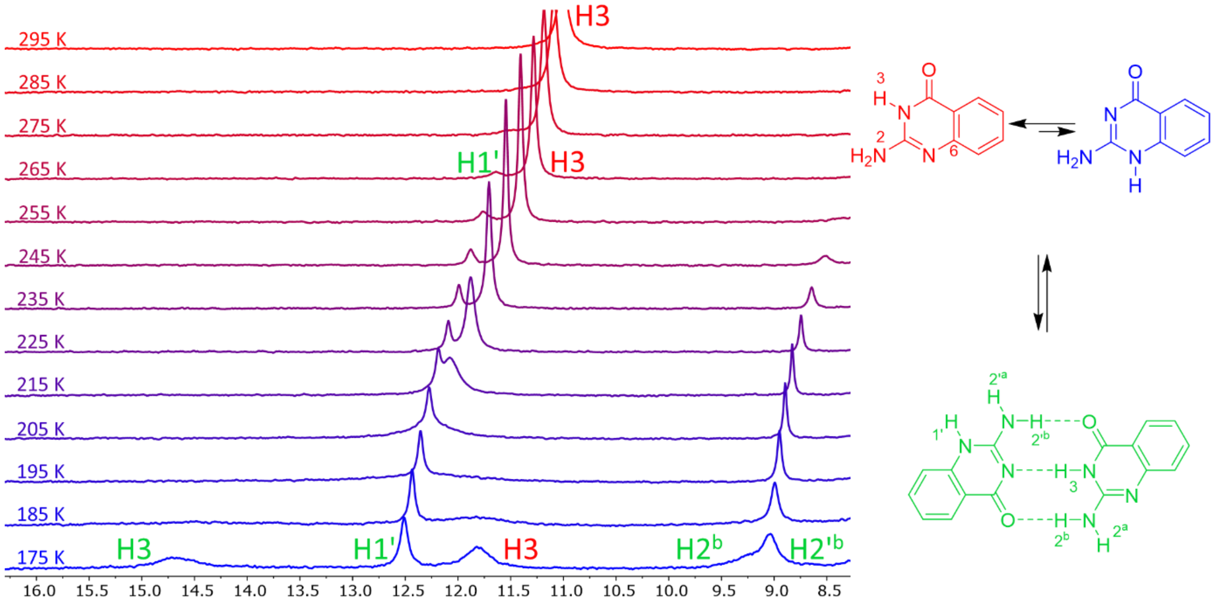

Figure S5. Low-field region of variable-temperature ^{1}H NMR spectra of compound **16** in a 3:1 mixture of DMF-*d*$_7$ and CD_2Cl_2.

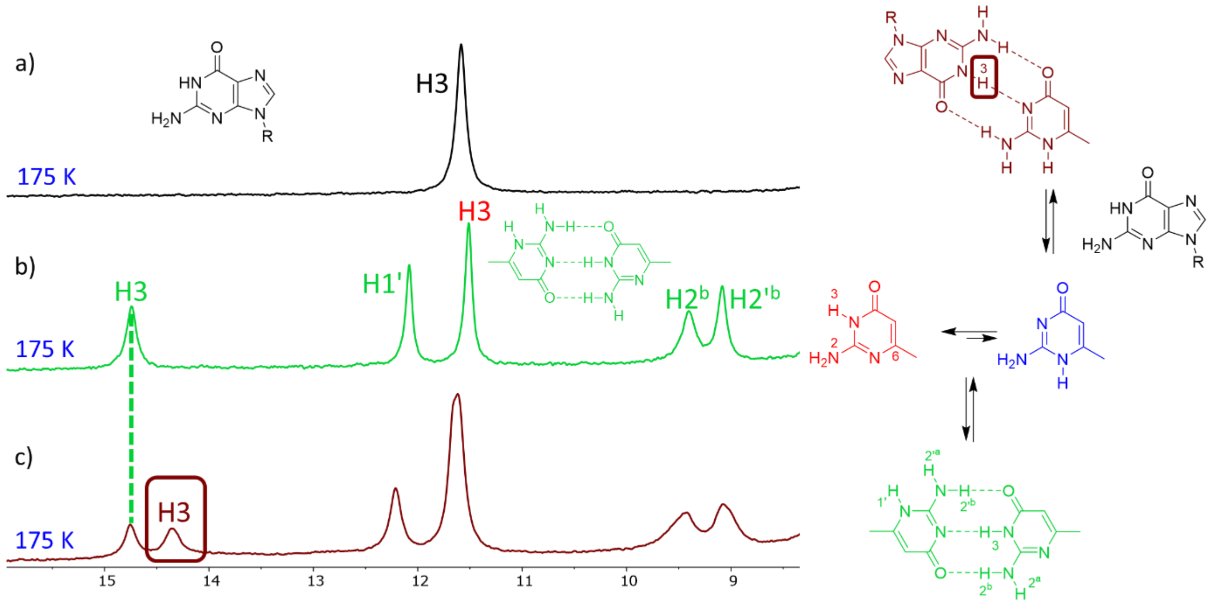

Figure S6. Low-field region of ^{1}H NMR spectra of compound **18** (a), compound **7** (b) and their 1:1 mixture (c).

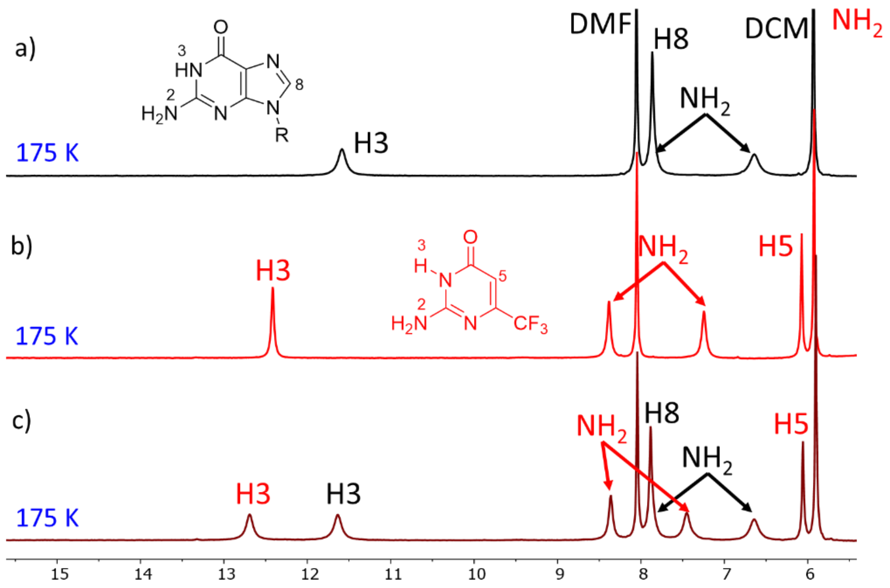

Figure S7. Low-field region of ^{1}H NMR spectra of compound **18** (a), compound **10** (b) and their 1:1 mixture (c).

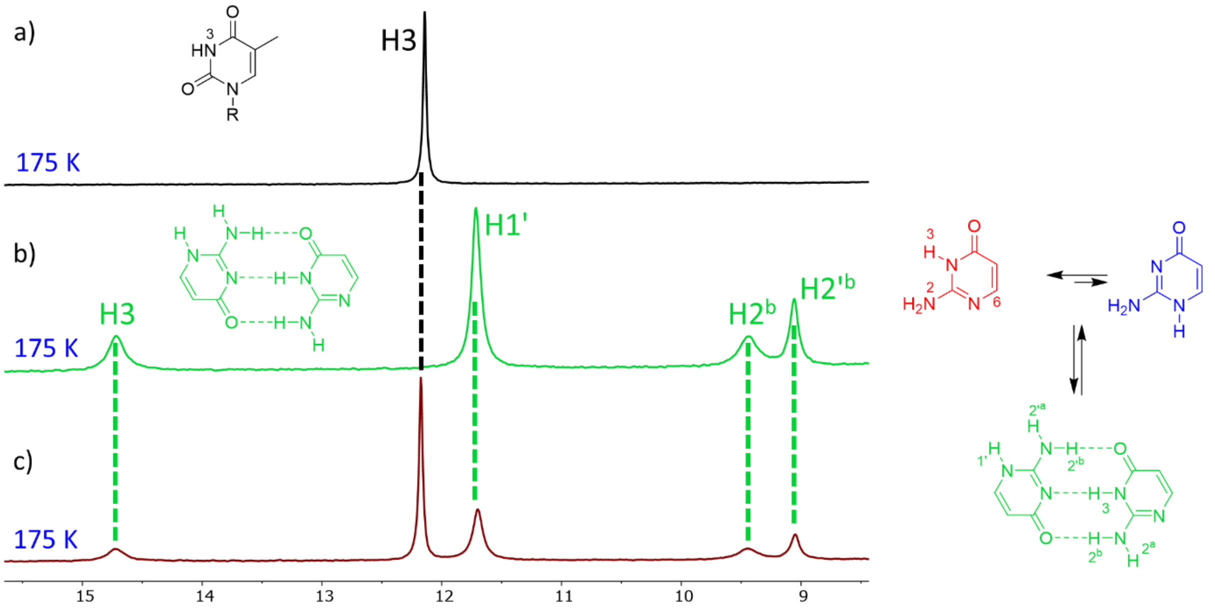

Figure S8. Low-field region of ^{1}H NMR spectra of compound **1** (a), compound **T** (b) and their 1:1 mixture (c).

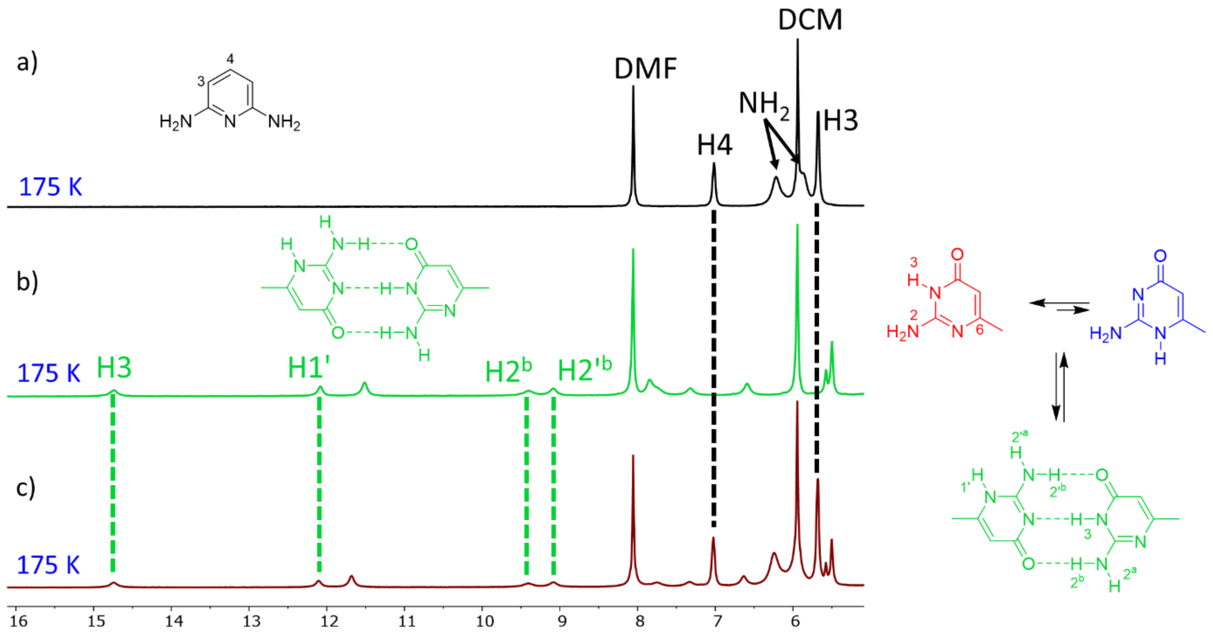

Figure S9. Low-field region of ^{1}H NMR spectra of compound **7** (a), compound **DAP** (b) and their 1:1 mixture (c).
